# Growth of tomato and cucumber seedlings under different light environments and their development after transplanting

**DOI:** 10.3389/fpls.2023.1164768

**Published:** 2023-07-18

**Authors:** Xiaojuan Liu, Rui Shi, Meifang Gao, Rui He, Yamin Li, Houcheng Liu

**Affiliations:** College of Horticulture, South China Agricultural University, Guangzhou, China

**Keywords:** light conditions, seedling quality, comprehensive evaluation, tomato, cucumber

## Abstract

Selecting suitable light conditions according to the plant growth characteristics is one of the important approaches to cultivating high-quality vegetable seedlings. To determine the more favorable LED light conditions for producing high-quality tomato and cucumber seedlings in plant factories with artificial light (PFALS), the growth characteristics of tomato and cucumber seedlings under seven LED light environments (CK, B, UV-A, FR, B+UV-A, UV-A+FR, and B+FR) and the development of these seedlings after transplanting into a plastic greenhouse were investigated. The results showed that the seedling height and hypocotyl length increased in treatments with far-red light supplementation (FR, UV-A+FR, and B+FR), but decreased in the B treatment, in both varieties. The seedling index of tomato seedlings increased in the B+UV-A treatment, while that of cucumber seedlings increased in the FR treatment. After transplanting into a plastic greenhouse, tomato plants that radiated with UV-A had greater flower numbers on the 15th day after transplanting. In cucumber plants of the FR treatment, the flowering time was significantly delayed, and the female flower exhibited at a lower node position. By using a comprehensive scoring analysis of all detected indicators, light environments with UV-A and FR were more beneficial for improving the overall quality of tomato and cucumber seedlings, respectively.

## Introduction

Seedling growth is fundamental to plant growth and important for vegetable yield and quality. Seedlings with poor quality often behave worse in terms of growth, development, yield, and quality after transplanting (Jeong et al., 2020). Therefore, it is necessary to adopt reasonable cultivation measures, such as changing the nutrient solution, temperature, carbon dioxide concentration, and so on, light environment, to improve the quality of vegetable seedlings on the basis of the growth requirements of different vegetables (Balliu, 2017).

Light, which can provide energy and regulate multiple biological processes, is vital for plants. Plant factories with artificial light (PFALs) are indoor farms, which have gradually developed into stable and large-scale vegetable seedling production and cultivation facilities (Kozai, 2018). APFAL is composed of a soilless culture system, an artificial lighting system, and environmental regulatory devices, among which the artificial lighting system plays an essential role (Kozai, 2013). Since the late 2000s, LEDs have been introduced into PFALs for artificial lighting and applied progressively due to their increased effectiveness and lower electricity costs (Goto, 2012). In contrast to traditional lights, LED lamps have a smaller size, higher stable radiation efficiency, greater environmental friendliness, and longer lifetimes (Sabzalian et al., 2014). Moreover, LED lights can conveniently and accurately regulate plant growth and development by adjusting the light environment (light quality, intensity, and photoperiod) (Morrow, 2008).

In recent years, a wide range of research has revealed the influences of various LED spectra on the growth and development of different vegetable seedlings. For monochromic light, compared with pea seedlings treated with white light, red light radiation led to significantly higher stem length and leaf area, with increased antioxidant activity, while blue light radiation induced increases in seedling weight and chlorophyll content (Wu et al., 2007). Under exposure to the orange light treatment, tomato seedlings showed a higher and weaker phenotype than those under the control (white light) treatment (Liu et al., 2012). Although cucumber seedlings treated with monochromic red, blue, yellow, and green light displayed inhibited plant growth and decreased chlorophyll content compared to the white light, each monochromatic light played a special role in the regulation of plant morphogenesis and photosynthesis (Su et al., 2013). Meanwhile, research on the effects of combined spectra on plant growth and development is increasing. Being effectively absorbed by chloroplasts, red and blue light lead to improved plant photosynthesis (Carvalho et al., 2010; Chen et al., 2021). The positive effects of these two light spectral combinations on plant growth and development have been revealed in many vegetable varieties, including tomato, cucumber, and pepper (Hernández and Kubota, 2016; Son et al., 2018; Li et al., 2020). In terms of other light spectrum combinations, the RGB (red + green + blue) pepper seedlings had greater seedling height, stem diameter, and total leaf area than seedlings of the GB (green + blue) treatments (Claypool and Lieth, 2020). Supplementary light with UV-A or far-red affects the morphology of cabbage and kale seedlings grown under pure blue light (B), with different responses to the same light conditions in these two varieties (Kong et al., 2019). Even so, further exploration is still needed to determine the positive impacts of various monochromatic lights and their different combinations on the growth and development of different kinds of vegetable seedlings.

Tomatoes and cucumbers are two of the world’s most popular fruit vegetables. With the enhancement of human awareness of healthy living, the demand for fresh vegetables (including tomatoes and cucumbers) continues to increase (Stanaway et al., 2022). Consequently, the demand for high-quality tomato and cucumber seedlings in the vegetable industry is also increasing. However, tomato and cucumber seedlings grown outdoors are easily affected by bad weather, poor soil quality, pests, etc., resulting in poor-quality seedlings, whereas those planted in plant factories are independent of these conditions (Kozai, 2013). To date, lots of investigations have reported the impacts of different LED light conditions on the growth and quality of tomato and cucumber seedlings (Nanya et al., 2012; Khoshimkhujaev et al., 2014; Hwang et al., 2020; Jeong et al., 2020; Hamedalla et al., 2022; Jin et al., 2023), but few of them have determined the relatively suitable LED light conditions for improving their seedling qualities in plant factories through comprehensive comparative analysis. Additionally, relatively few studies have displayed the effects of different light conditions on the post-transplantation growth performance of tomato and cucumber seedlings. Furthermore, the growth responses of tomato and cucumber seedlings to different combinations of different light qualities are still not fully known.

In this study, under conditions of similar light intensity (photosynthetic photon flux density, PPDF of 250 μmolm^−2^ s^−1^), the various growth indices of tomato and cucumber seedlings supplemented with different LED light spectra (blue, UV-A, and far-red) and these light spectrum combinations, as well as the growth performance of these seedlings after transplant into a plastic greenhouse, were examined. A formula was established to comprehensively compare and analyze all measured indices, in order to select the more favorable LED light conditions for improving the growth quality of tomato and cucumber seedlings. These results will provide a theoretical basis for cultivating tomato and cucumber seedlings with high uniformity and quality in plant factories by altering the light environments.

## Materials and methods

### Plant material and growth conditions

**Figure 1:**
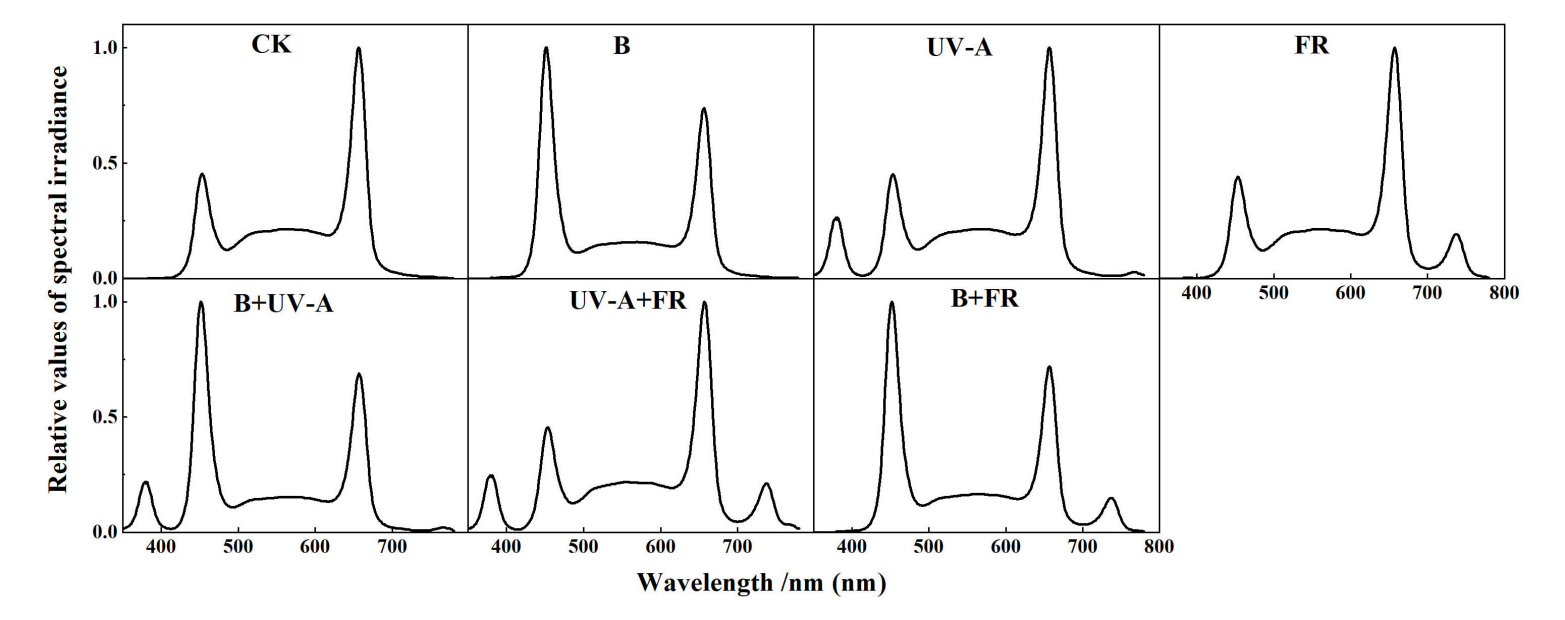
The light spectral of different light treatments used in this experiment.

All the seedlings in this experiment were grown in an indoor plant factory at South China Agricultural University (Guangzhou, China). Seeds of tomato (cv. Zhuanhong No. 2, Hunan Xingshu Seed Industry Co. Ltd.) and cucumber (cv. Zaoqing No. 2, Guangdong Kenong Vegetable Seed Industry Co. Ltd.) were sown in sponges of size 2 cm × 2 cm × 2 cm and pretreated under PPFD of 200 μmol m^−2^ s^−1^ white light for 2 days after germination. In the preliminary research, we discovered that white: red = 3:2 is superior to single white light for tomato and cucumber breeding. Tomato and cucumber seedlings were planted in the hydroponic systems after germination and divided into seven groups for different light treatments (CK: 3W2R (W:R=3:2); B: 3W2R+50 μmolm^−2^ s^−1^ B; UV-A: 3W2R+6 μmolm^−2^ s^−1^ UV-A; FR: 3W2R+30 μmolm^−2^ s^−1^FR; B+UV-A: 3W2R+50 μmolm^−2^ s^−1^ B+6 μmolm^−2^ s^−1^ UV-A; UV-A+FR: 3W2R+6 μmolm^−2^ s^−1^ UV-A +30 μmolm^−2^ s^−1^ FR; B+FR: 3W2R+50 μmolm^−2^ s^−1^ B+30 μmolm^−2^ s^−1^ FR), respectively. The light sources were provided by stable and adjustable LED panels (Chenghui Equipment Co., Ltd., Guangzhou, China; 150 cm × 30 cm). The seedling growth conditions were set to 24 ± 2°C, 75 ± 5% relative humidity (RH). All the seedlings were grown under a similar PPFD of 250 μmol m^−2^ s^−1^, with a 12/12 h (light/dark) duration, and supplied with a 1/2 Hoagland solution (pH 5.5; EC, 1.30 mS cm^−2^). The respective light intensities of the PPFD and light spectra (W: 400–700 nm; R: 660 ± 10 nm; B: 450 ± 10 nm, UV-A: 385 ± 10 nm, FR: 735 ± 10 nm) were determined by using the APL-01 machine (Asensetek, Taiwan) (**Figure 1**; **Supplementary Table S1**). To ensure the accuracy of light intensity and quality, we measured them twice a day using the APL-01 machine during plant treatment, once in the morning and once in the afternoon, respectively. After 15 days of light treatments, all the seedlings were transplanted into a plastic greenhouse (South China Agricultural University, Guangzhou, China).

### Plant growth analysis and biomass determination

After 15 days of light treatment, 15 uniform seedlings of tomato and cucumber were selected to determine their growth indices. A measuring ruler was used to measure the seedling height (cm) and hypocotyl length (cm). The stem diameter (mm) was detected by using a vernier caliper.

The true leaf number of all treated seedlings was counted. The total leaf area (cm^2^) of tomato and cucumber seedlings was measured using a leaf area meter (LI-3000A). The fresh weight (g/plant) was measured with an electronic balance (BCE224-1CCN, Sartorius, Beijing), and the dry weight (g/plant) was determined after drying at 105°C for 30 min and then dried at 75°C to a constant weight.

### Determination of comprehensive seedling indices

The following formulas were used to calculate the comprehensive seedling indexes of tomato and cucumber seedlings:

Specific leaf weight = leaf dry weight/leaf area,

Plant compactness = shoot dry weight/plant height,

Root shoot ratio = root dry weight/shoot dry weight,

The seedling index = (stem diameter/plant height + root dry weight/shoot dry weight) * whole plant dry weight (Bai et al., 2014).

### Determination of photosynthetic pigment content and chlorophyll fluorescence parameters

A total of 0.5 g fresh samples of tomato and cucumber seedlings was taken for photosynthetic pigment content (chlorophyll and carotenoids) determination. In brief, the sample was placed in the dark at room temperature with 8 ml of an acetone–alcohol mixture (acetone: alcohol = 1:1, v/v) overnight until it turned white. A UV spectrophotometer (Shimadzu UV-16A, Shimadzu, Corporation, Kyoto, Japan) was used to obtain the absorbance of the supernatant at 440, 645, and 663 nm. Chlorophyll a (mg/g), chlorophyll b (mg/g), total chlorophyll (mg/g), and carotenoid content (mg/g) were calculated according to the methods described by He et al. (He et al., 2021).

Using the third true leaf of each seedling as a sample, measurements of chlorophyll fluorescence parameters were performed using a fluorometer (MINI-PAM-II, Germany).

### Evaluation of phytochemical substance and enzyme activities of seedlings

Following the experimental steps reported in He et al. (He et al., 2021), the total soluble protein content was determined by the Coomassie brilliant blue G-250 dye method. To detect the activities of the antioxidant enzymes [superoxide dismutase (SOD), peroxidase (POD), and catalase (CAT)], 0.5 g fresh leaf from seedlings was ground in 10 ml phosphate buffer (50 mM, pH 7.8) and centrifuged at 10,000 rpm for 20 min; then, the supernatant was collected and used for further analysis. The reaction solution consisted of 50 mM phosphate buffer, 13 mM methionine, 2 μM riboflavin, 10 μM EDTA-Na_2_, 75 μM NBT, and 50 μl enzyme extract. The SOD activity was estimated at 560 nm following the method of Li and Yi (2012). The reaction mixture measuring POD activity contained 0.8 ml enzyme extract, 1.45 ml phosphate buffer, 0.5 ml guaiacol (50 mM/L), and 0.5 ml H_2_O_2_ (2%), and was determined at 470 nm. Changes in absorbance at 0.01 units min^−1^ were defined as one unit of POD activity (Raza et al., 2007). For CAT activity, the reaction mixture contained 0.2 ml enzyme extraction, 1.5 ml phosphate buffer, 1 ml distilled water, and 200 mM H_2_O_2_. By measuring at 240 nm, one unit of CAT activity was expressed as 0.1 units min^−1^ change in absorbance (Seckin et al., 2008).

To access the malondialdehyde (MDA) content, a total of 0.5 g fresh sample was homogenized in a 10-ml 10% trichloroacetic acid (TCA) solution. After centrifugation (4,000 rpm, 10 min), the supernatant was subjected to a further reaction. The reaction solution (contained 2 ml supernatant and 2 ml 0.6% TBA solution) was heated at 100°C for 15 min, followed by rapid cooling, and then centrifuged at 4,000 rpm for 10 min. After that, the supernatant was measured at 532, 600, and 450 nm using a UV spectrophotometer (Zhang et al., 2005).

### Growth analysis of plant performance after transplanting

After 15 days of light treatment, 24 tomato and cucumber seedlings with uniform growth from each treatment were transplanted into a plastic greenhouse. All the seedlings were grown in coir tanks and fertilized with a drip irrigation system using the Yamazaki nutrient solution. For tomato plant growth indicators within 30 days after transplanting (DAT), the flowering time, the node position of the first flower, and the number of fruits per plant at 15 and 30 DAT were recorded. For cucumber plants of 20 DAT, growth parameters, including the flowering time, the node position of the first flower, the node position of the first female flower, the number of female flowers, and the total number of flowers within 15 nodes, were counted.

### Statistical analysis

All statistical analyses were performed by using SPSS 26.0 and Origin 2021. The Duncan’s multiple range test was used to determine the significant differences at the 0.05 significance level (*p* < 0.05) using SPSS 26.0. The graphing was conducted by Origin 2021.

## Results

### Impacts of diverse LED light qualities on the morphology and biomass of vegetable seedlings

**Figure 2:**
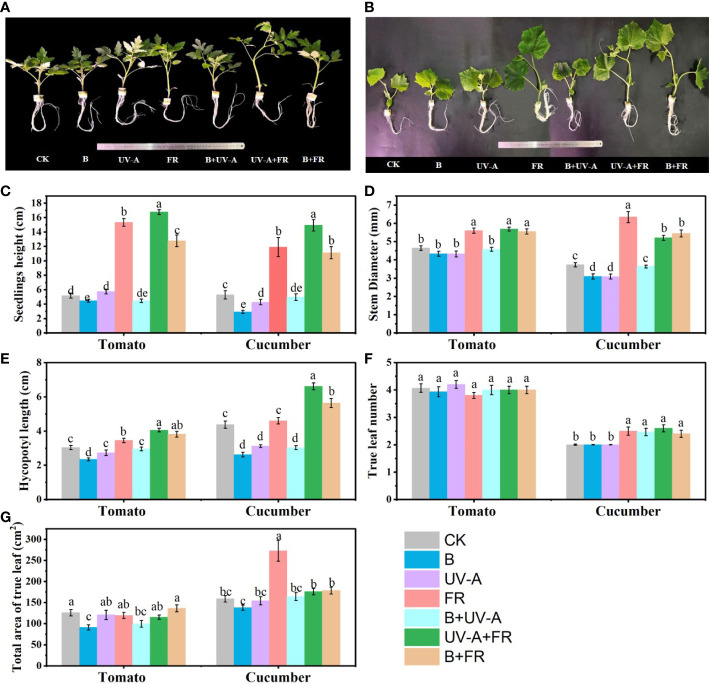
Influence of various light treatments on the growth of the tomato and cucumber seedlings. The morphology of **(A)** tomato seedlings and **(B)** cucumber seedlings cultivated under different light environments for 15 days. **(C)** The seedling height, **(D)** stem diameter, **(E)** hypocotyl length, **(F)** true leaf number, and **(G)** total leaf area of tomato and cucumber seedlings after light treatments. Data represent mean ± SE followed by the same letters do not differ significantly according to the Duncan’s multiple range test.

Obviously, different LED light conditions had significant effects on the morphology of tomato and cucumber seedlings (**Figures 2A, B**). In the two species, the FR, UV-A+FR, and B+FR treatments significantly promoted seedling growth, including seedling height, stem diameter, and hypocotyl length (**Figures 2A, B**). Specifically, compared to the control, the seedling height, stem diameter, and hypocotyl diameter of tomato seedlings were increased by more than 102%, 19.0%, and 13.0%, respectively, while those in cucumber increased by more than 70%, 45.0%, and 17.0%, respectively (**Figures 2C–E**). However, the seedling height and hypocotyl length of cucumber and tomato seedlings significantly decreased in the B treatment (**Figures 2C–E**). In the UV-A treatment, the seedling height, hypocotyl length, and stem diameter of tomato seedlings were not affected, but those of cucumber seedlings obviously decreased (**Figures 2C–E**). Compared with the control, the B+UV-A treatment led to a noticeable decrease in the height of tomato seedlings and the hypocotyl length of cucumber seedlings, respectively (**Figures 2C–E**). In the aspects of leaf development, the true leaf number of tomato seedlings exhibited no significant difference among all treatments, while that of cucumber seedlings in the UV-A, FR, UV-A+FR, and B+FR treatments significantly increased (**Figure 2F**). The total leaf area of tomato seedlings grown under the B and B+UV-A treatments was 27.6% and 20.9% lower than those of seedlings grown under the control, respectively (**Figure 2G**). Compared to the control of cucumber seedlings, the total leaf area was the highest in the FR treatment, while no significant difference was found among the other treatments (**Figure 2G**).

**Figure 3:**
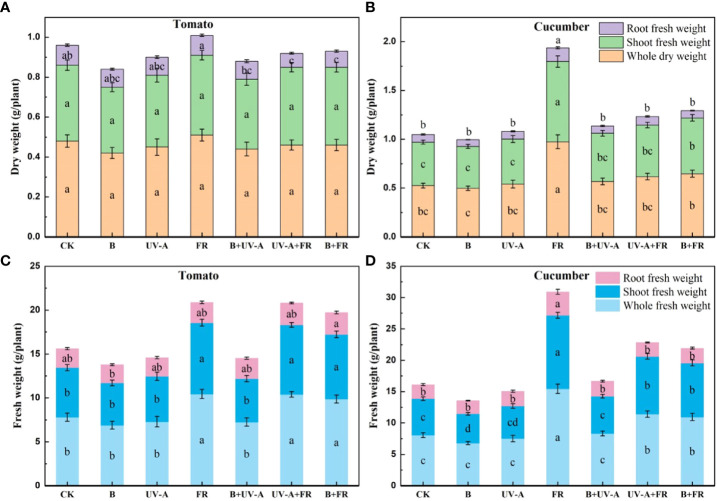
Changes of biomass in tomato and cucumber seedlings treated by different light environments. The dry weight of **(A)** tomato seedlings and **(B)** cucumber seedlings and **(C)** the fresh weight of tomato seedlings and **(D)** cucumber seedlings cultivated under different light environments. Data represent mean ± SE followed by the same letters do not differ significantly according to the Duncan’s multiple range test.

As shown in **Figure 3**, the dry weight of the plant and shoot exhibited no significant difference among all the treatments in tomato seedlings (**Figure 3A**). Except for the significant reduction in the UV-A+FR and B+FR treatments, there was no significant difference in root dry weight of tomato seedlings between the other treatments and the control (**Figure 3A**). When irradiated under the FR treatment, cucumber seedlings exhibited a markable increase in the dry weight of the plant, shoot, and root (**Figure 3B**). In both varieties, the fresh weight of plant and shoot in the FR, UV-A+FR, and B+FR treatments was obviously elevated (**Figure 3C**). The cucumber seedlings in the B treatment showed a reduction in the shoot fresh weight, whereas those in the UV-A and B+UV-A treatments did not significantly differ from the control (**Figure 3D**). No significant difference was found in the root fresh weight among all the treatments in tomato seedlings (**Figure 3D**). The root fresh weight of tomato seedlings in the FR treatment was significantly higher than that in the control treatment (**Figure 3D**).

### Influences of diverse LED light qualities on the comprehensive indices of seedling morphology

The effects of distinct LED light environments on comprehensive indices of seedling morphology were analyzed. In tomato seedlings, the plant compactness in the B and B+UV-A treatments was significantly higher than that in the control, whereas it was significantly decreased in the FR, UV-A+FR, and B+FR treatments (**Table 1**). The trend of alternation in plant compactness in cucumber seedlings under different light conditions followed the same pattern as that in tomato seedlings (**Table 1**). Tomato seedlings exposed to different LED light treatments showed no comparable difference in the root shoot ratio, while in cucumber, FR treatment induced a significant decrease in the root shoot ratio compared to the control, which decreased by 37.1% (**Table 1**). When compared to the control, the specific leaf weight of tomato seedlings was clearly increased in the B and B+UV-A treatments but decreased in the UV-A+FR and B+FR treatments (**Table 1**). Cucumber seedlings in the FR, B+UV-A, and B+FR treatments showed obviously elevated specific leaf weight in comparison with the control (**Table 1**). For tomato seedlings, the highest seedling index was observed in the B+UV-A treatment, while the lowest occurred in the UV-A+FR treatment (**Table 1**). As for cucumber seedlings, the seedling index in the FR treatment was the highest, increasing by 54.1% compared with the control, although there was no significant difference between the other treatments and the control (**Table 1**).

**Table 1:** Comprehensive growth indexes of tomato and cucumber seedlings cultivated under different LED light environments.

Species	Treatment	Compactness	Root shoot ratio	Specific leaf weight	The seedling index
Tomato	CK	42.41 ± 2.87 b	0.28 ± 0.02 a	2.28 ± 0.080 b	0.35 ± 0.02 b
	B	50.39 ± 2.17 a	0.27 ± 0.01 a	3.40 ± 0.301 a	0.41 ± 0.02 ab
	UV-A	46.29 ± 3.30 ab	0.25 ± 0.01 a	2.39 ± 0.057 b	0.40 ± 0.03 ab
	FR	19.87 ± 0.91 c	0.26 ± 0.01 a	2.53 ± 0.158 b	0.26 ± 0.01 c
	B+UV-A	53.01 ± 3.06 a	0.24 ± 0.01 a	3.18 ± 0.278 a	0.43 ± 0.03 a
	UV-A+FR	18.52 ± 0.81 c	0.18 ± 0.01 b	1.98 ± 0.099 c	0.21 ± 0.01 c
	B+FR	22.54 ± 1.31 c	0.20 ± 0.01 b	1.98 ± 0.091 c	0.25 ± 0.02 c
Cucumber	CK	48.07 ± 3.26 c	0.94 ± 0.07 ab	2.09 ± 0.06 c	0.31 ± 0.03 bc
	B	77.95 ± 2.23 a	1.01 ± 0.04 a	2.37 ± 0.05 abc	0.36 ± 0.02 b
	UV-A	61.93 ± 2.43 b	1.00 ± 0.08 a	2.22 ± 0.08 bc	0.32 ± 0.02 bc
	FR	40.44 ± 3.71 d	0.59 ± 0.04 c	2.43 ± 0.07 ab	0.48 ± 0.05 a
	B+ UV-A	62.59 ± 2.66 b	0.90 ± 0.05 ab	2.61 ± 0.21 a	0.35 ± 0.02 b
	UV-A+FR	24.43 ± 0.77 e	0.86 ± 0.05 ab	2.32 ± 0.07 abc	0.25 ± 0.01 c
	B+FR	34.41 ± 1.51 d	0.78 ± 0.04 b	2.50 ± 0.08 ab	0.30 ± 0.02 bc

Data represent mean ± SE(n=20). Different letters indicate significant differences between treatments at the *p* < 0.05 using the Duncan’s test. Plant compactness = shoot dry weight/plant height, specific leaf weight = leaf dry weight/leaf area, root shoot ratio = root dry weight/shoot dry weight, the seedling index = (stem diameter/plant height + root dry weight/shoot dry weight) × whole plant dry weight.

### Effects of different LED light qualities on photosynthetic pigment content and chlorophyll fluorescence parameters

In tomato, when compared to the control, the UV-A+FR and B+FR treatments reduced the contents of chlorophyll a, chlorophyll b, and total chlorophyll, but in other treatments, there was no significant difference in these three pigments (**Table 2**). The carotenoid content of tomato seedlings did not differ among all the treatments in our experiment (**Table 2**). As regards cucumber seedlings, a significant higher content of chlorophyll a and total chlorophyll was observed only in the B treatment (**Table 2**). The chlorophyll b content of all cucumber leaves was not obviously affected by different light treatments (**Table 2**). The carotenoid content decreased by 15.0% in the B+FR treatment compared to the control (**Table 2**).

**Table 2:** The photosynthetic pigment content and photosynthetic characteristics of tomato and cucumber seedlings grown under various light environments.

Species	Treatment	Chlorophyll a (mg/g)	Chlorophyll b (mg/g)	Total Chlorophyll (mg/g)	Carotenoids (mg/g)	Fv/Fm	Y(II)	ETR
Tomato	CK	1.19 ± 0.01 a	0.53 ± 0.03 ab	1.74 ± 0.04 ab	0.04 ± 0.01 abc	0.79 ± 0.00 b	0.57 ± 0.00 a	13.24 ± 3.00 a
	B	1.21 ± 0.01 a	0.57 ± 0.04 ab	1.80 ± 0.05 ab	0.03 ± 0.01 bc	0.78 ± 0.00 b	0.50 ± 0.00 b	11.85 ± 6.03 b
	UV-A	1.22 ± 0.00 a	0.59 ± 0.01 a	1.83 ± 0.01 a	0.02 ± 0.00 c	0.78 ± 0.00 b	0.45 ± 0.00 b	10.50 ± 2.00 b
	FR	1.16 ± 0.03 abc	0.49 ± 0.03 bc	1.67 ± 0.06 bc	0.06 ± 0.01 ab	0.79 ± 0.00 b	0.50 ± 0.00 b	11.60 ± 3.00 b
	B+ UV-A	1.18 ± 0.01 ab	0.51 ± 0.01 abc	1.72 ± 0.02 abc	0.06 ± 0.01 ab	0.80 ± 0.00 a	0.51 ± 0.00 b	11.72 ± 3.00 b
	UV-A+FR	1.11 ± 0.04 c	0.44 ± 0.04 c	1.58 ± 0.07 c	0.08 ± 0.01 a	0.78 ± 0.00 b	0.51 ± 0.00 b	11.68 ± 3.00 b
	B+FR	1.13 ± 0.00 bc	0.44 ± 0.00 c	1.59 ± 0.01 c	0.07 ± 0.00 a	0.78 ± 0.00 b	0.51 ± 0.00 b	11.77 ± 5.00 b
Cucumber	CK	1.73 ± 0.01 bcd	0.55 ± 0.01 ab	2.31 ± 0.02 bcd	0.29 ± 0.00 a	0.77 ± 0.00 bc	0.40 ± 0.01 c	9.20 ± 0.30 c
	B	1.93 ± 0.03 a	0.62 ± 0.01 a	2.58 ± 0.04 a	0.28 ± 0.01 a	0.78 ± 0.00 ab	0.51 ± 0.01 a	11.80 ± 0.29 a
	UV-A	1.72 ± 0.03 cd	0.54 ± 0.01 b	2.28 ± 0.04 cd	0.30 ± 0.01 a	0.77 ± 0.00 c	0.47 ± 0.01 b	10.81 ± 0.27 b
	FR	1.70 ± 0.05 cd	0.52 ± 0.02 b	2.24 ± 0.07 cd	0.30 ± 0.00 a	0.75 ± 0.01 d	0.49 ± 0.00 ab	11.38 ± 0.08 ab
	B+ UV-A	1.90 ± 0.09 ab	0.63 ± 0.05 a	2.56 ± 0.14 ab	0.28 ± 0.01 a	0.77 ± 0.00 bc	0.46 ± 0.01 b	10.59 ± 0.35 b
	UV-A+FR	1.61 ± 0.06 d	0.52 ± 0.02 b	2.16 ± 0.08 d	0.29 ± 0.00 a	0.77 ± 0.00 bc	0.46 ± 0.01 b	10.72 ± 0.23 b
	B+FR	1.84 ± 0.06 abc	0.60 ± 0.03 ab	2.47 ± 0.09 abc	0.25 ± 0.00 b	0.79 ± 0.00 a	0.51 ± 0.01a	11.85 ± 0.24 a

Data represent mean ± SE (n=20). Different letters indicate significant differences between treatments at the *p* < 0.05 using the Duncan’s test.

For tomato, the Fv/Fm ratio significantly increased under the B+UV-A treatment, while no difference was found between the other treatments and the control (**Table 2**). In cucumber seedlings, the Fv/Fm value significantly increased in the B+FR treatment, but there was a decrease in the FR treatment compared with the control (**Table 2**). Interestingly, compared with the control, both the Y(II) and electron transportation rate (ETR) values of the other treatments in tomato seedlings decreased (**Table 2**), while in cucumber seedlings, the opposite trend was observed, showing that Y(II) and ETR values were the lowest in the control (**Table 2**).

### Influences of different LED light qualities on physiological characteristics and antioxidant enzyme activities of vegetable seedlings

**Figure 4:**
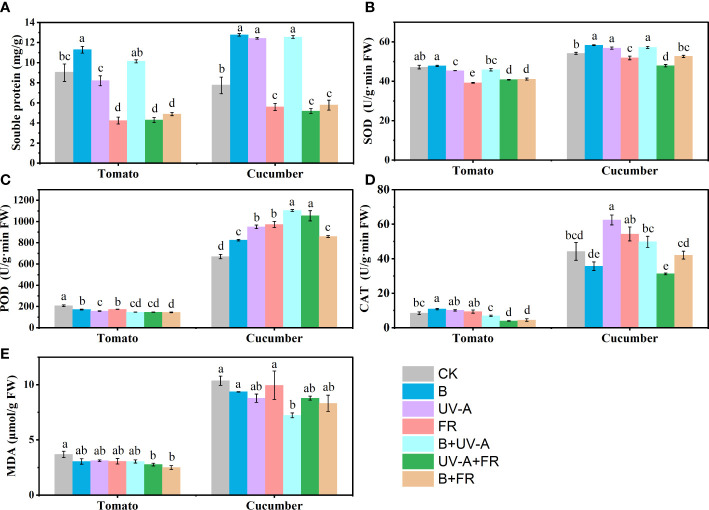
The differences in physiological characteristics and antioxidant enzyme activities in tomato and cucumber seedlings under different light conditions. **(A)** The soluble protein content, activity of SOD **(B)**, POD **(C)**, CAT **(D)**, and MDA content **(E)** in tomato and cucumber seedlings after 15 days of light treatments. Data represent mean ± SE followed by the same letters do not differ significantly according to the Duncan’s multiple range test.

In tomato seedlings, compared with the control, the soluble protein content was significantly higher in the B treatment, whereas it was significantly lower in the FR, UV-A+FR, and B+FR treatments (**Figure 4A**). Seedlings in the UV-A, FR, B+UV-A, UV-A+FR, and B+FR treatments showed a marked decrease in the activity of SOD compared to the control (**Figure 4B**). Relative to the control, a significant decrease in the POD activity was observed in all the other treatments (**Figure 4C**). In contrast to the control, the CAT activity increased in the B treatment, whereas it decreased in the UV-A+FR and B+FR treatments (**Figure 4D**). The MDA content was unaffected in the B, UV-A, FR, and B+UV-A treatments but decreased in the UV-A+FR and B+FR treatments (**Figure 4E**).

In the case of cucumber, the B, UV-A, and B+UV-A treatments led to noticeable increases in the soluble protein content compared with the control, whereas the soluble protein content significantly decreased in the FR, UV-A+FR, and B+FR treatments, respectively (**Figure 4A**). The B, UV-A, and B+UV-A treatments increased, while the UV-A+FR and B+FR treatments markedly decreased the activity of SOD when compared to the control (**Figure 4B**). In comparison with the control, increments in the activity of POD were noted under all the other treatments (**Figure 4C**). The highest activity of CAT was detected in the UV-A treatment, while the lowest occurred in the UV-A+FR treatment (**Figure 4D**). Seedlings grown under the B+UV-A treatment had the highest MDA content, while no significant difference was found between the other treatments and the control (**Figure 4E**).

### Effects of different LED light conditions on transplant development of different seedlings

We further assessed the growth and development of these plants treated differently after transplantation. In tomato, the flowering time of all the plants showed no notable difference under different light conditions, whereas it tended to be later in the B+UV-A, UV-A+FR, and B+FR treatments than in the UV-A treatment (**Table 3**). The first flower in seedlings treated with UV-A+FR and B+FR exhibited at higher node position, while no significant difference was observed between the other treatments and the control (**Table 3**). On the 15th DAT, the flower number of plants treated in the UV-A treatment was the highest (**Table 3**). The total fruit number of plants on the 30th DAT was not significantly affected by various LED light treatments (**Table 3**).

**Table 3:** Performance of transplant tomato plants treated with different light conditions at the seedling stage.

Species	Treatment	Time to first flower (day)	Node position of the first flower	Flower numbers at the 15th DAT	Fruit numbers at the 30th DAT
Tomato	CK	16.07 ± 0.27 ab	8.13 ± 0.09 cd	2.13 ± 0.19 b	2.07 ± 0.38 a
	B	16.60 ± 0.27 ab	8.27 ± 0.12 bc	2.20 ± 0.30 b	1.47 ± 0.41 a
	UV-A	15.87 ± 0.13 b	7.87 ± 0.09 d	3.13 ± 0.22 a	1.87 ± 0.34 a
	FR	16.60 ± 0.29 ab	8.47 ± 0.13 abc	1.87 ± 0.38 b	1.20 ± 0.24 a
	B+ UV-A	16.73 ± 0.28 a	7.87 ± 0.13 d	2.47 ± 0.34 ab	1.87 ± 0.39 a
	UV-A+FR	16.80 ± 0.28 a	8.53 ± 0.13 ab	1.60 ± 0.31 b	1.53 ± 0.42 a
	B+FR	16.80 ± 0.26 a	8.73 ± 0.12 a	2.00 ± 0.29 b	1.00 ± 0.32 a

Data represent mean ± SE (n=20). Different letters indicate significant differences between treatments at the *p* < 0.05 using the Duncan’s test.

In cucumber plants, compared with the control, plants exposed to the UV-A and FR treatments flowered significantly earlier, but these was not affected in the other treatments (**Table 4**). The node position of the first flower did not differ among all the treatments (**Table 4**). However, on the 20th DAT, the node position of the first female flower in the FR treatment was the lowest, which is significantly different from the control (**Table 4**). In particular, the total flower number of seedlings grown in the UV-A+FR and B+FR treatments was clearly lower than that in the control (**Table 4**).

**Table 4:** Growth of cucumber seedlings grown under distinct light treatments after transplanting.

Species	Treatment	Time to first flower (day)	Node position of the first flower	At the 20th DAT (within 15 nodes)		
				Node position of the first female flower	Female flower numbers	Total flower numbers
Cucumber	CK	34.7 ± 0.2 ab	4.4 ± 0.5 a	6.3 ± 0.5 ab	2.0 ± 0.4 a	4.3 ± 0.4 a
	B	34.1 ± 0.2 bc	3.5 ± 0.4 a	6.4 ± 0.5 ab	1.5 ± 0.4 a	4.2 ± 0.3 ab
	UV-A	31.9 ± 0.1 d	3.5 ± 0.4 a	6.1 ± 0.7 abc	1.2 ± 0.4 a	4.2 ± 0.4 ab
	FR	33.9 ± 0.3 c	3.1 ± 0.4 a	4.7 ± 0.5 c	1.5 ± 0.3 a	3.9 ± 0.4 ab
	B+ UV-A	34.4 ± 0.2 abc	4.5 ± 0.7 a	7.3 ± 0.4 a	1.7 ± 0.2 a	4.1 ± 0.4 ab
	UV-A+FR	34.1 ± 0.3 bc	3.7 ± 0.3 a	5.2 ± 0.3 bc	1.6 ± 0.3 a	3.1 ± 0.3 bc
	B+FR	34.9 ± 0.1 a	4.1 ± 0.3 a	5.0 ± 0.4 bc	1.2 ± 0.3 a	2.5 ± 0.4 c

Data represent mean ± SE (n=20). Different letters indicate significant differences between treatments at the *p* < 0.05 using the Duncan’s test.

### Correlation analysis and comprehensive analysis

The Pearson correlation and principal component analysis (PCA) were performed to analyze the correlation between each examined growth index, and a formula was constructed to comprehensively analyze the growth quality of vegetable seedlings under different light environments.

#### Tomato seedlings

**Figure 5:**
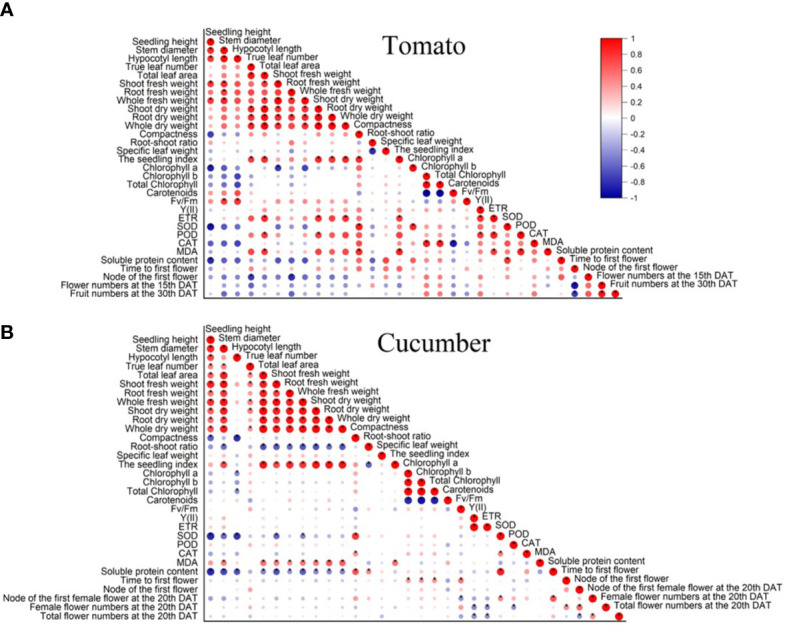
The Pearson correlation of **(A)** tomato and **(B)** cucumber seedlings under different light conditions. All examined indexes in each variety were involved in the analysis. * means significance level (*p* < 0.05).

According to the results of Pearson correlation, a significant positive correlation was exhibited between the seedling index and compactness, true leaf number, total leaf area, whole dry weight, root dry weight, shoot dry weight, whole fresh weight, ETR, and MDA content, whereas no significant negative correlation was found between the seedling index and all of the examined indicators (**Figure 5A**).

**Figure 6:**
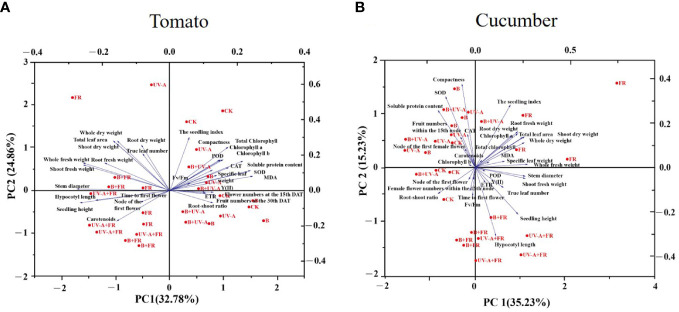
PCA analysis of **(A)** tomato and **(B)** cucumber seedlings grown under various light conditions.

Similarly, the results of PCA revealed that the seedling index was positively related to true leaf number, total leaf area, whole dry weight, shoot dry weight, root dry weight, and plant compactness, whereas it is negatively correlated with stem diameter, seedling height, hypocotyl length, and carotenoid content (**Figure 6A**). In addition, as can be seen, the seedling growth difference between the FR, UV-A+FR, and B+FR treatments was small. Analogously, a relatively small difference in seedling growth existed between the B, UV-A, B+UV-A, and CK treatments (**Figure 6A**).

To simplify these indices, OriginPro 2021 software was used to build a formula to simplify these evaluated indicators. Then, we constructed a comprehensive score model for comprehensively evaluating the performance of tomato seedlings grown under various light conditions. With reference to the methods of Huang et al. (2021) and Sabzalian et al. (2014), the formula was Y = 32.78% Y1+24.86% Y2 (**Supplementary Table S2**). Using this formula, the optimal light treatment for the growth and quality of tomato seedlings was UV-A > B > CK > B+UV-A > FR > B+FR > UV-A+FR.

#### Cucumber seedlings

In cucumber seedlings, the seedling index was positively correlated with the stem diameter, true leaf number, total leaf area, whole fresh weight, shoot fresh weight, root fresh weight, shoot dry weight, root dry weight, and MDA content (**Figure 5B**). However, it was negatively correlated with the root shoot ratio and had no significant correlation with the other examined indicators (**Figure 5B**).

As was shown, following the PCA, the seedling index was positively related with chlorophyll a, total leaf area, whole dry weight, shoot dry weight, root dry weight, whole fresh weight, root fresh weight, and MDA content (**Figure 6B**). In terms of growth performance, seedlings grown in the CK, B, UV-A, and B+UV-A treatments showed small growth differences, while the growth of those seedlings was significantly different from that in the UV-A+FR and B+FR treatments (**Figure 6B**).

By means of the above-described method, Y=35.23% Y1+15.23% Y2 was the calculated formula of the comprehensive score model (**Supplementary Table S3**). According to this model, the optimal light quality for the growth and quality of cultivated cucumber seedlings was as follows: FR > UV-A+FR > B > B+UV-A > UV-A > B+FR > CK.

## Discussion

### Effects of different light qualities on the morphology and biomass of tomato and cucumber seedlings

The negative role of blue light in plant height has been previously described in many species, such as tomato, cucumber, and pepper (Nanya et al., 2012; Hamedalla et al., 2022; Liu et al., 2022). In this study, we also obtained similar results (**Figure 2C**). Adding 6.8 W-m^−2^ UV-A to monochromatic red light significantly reduced the height of tomato seedlings (Khoshimkhujaev et al., 2014). Differently, our data displayed that supplemental radiation of 6 μmol m^−2^ s^−1^ UV-A to the CK (white: red= 3:2) had no significant effect on the seedling height of tomato (**Figure 2C**). These might be due to the different responses of plants to different combinations of light wavelengths. However, unlike in tomato, supplementing with 6 μmol m^−2^ s^−1^ UV-A significantly inhibited the height of cucumber seedlings (**Figure 2C**), suggesting that the response of seedling height to supplemental UV-A light might vary in different vegetable varieties. When plant cryptochromes perceive UV-A and blue radiations, the synthesis or sensitivity of gibberellin and auxin in plants is affected, resulting in stem elongation inhibition (Huchéthélier et al., 2016). However, in both tomato and cucumber, seedlings in the UV-A+FR and B+FR treatments exhibited higher seedling height and hypocotyl length than those in the control (**Figure 2C**), meaning that when combined with blue or UV-A light, far-red light played a predominant role in the effect of seedling height and hypocotyl length. When exposed to far-red light, the increased seedling height in both tomato and cucumber might be due to the reduction in the R:FR ratio caused by supplementing far-red light, which is sensed by phytochrome and induces a shade-avoidance syndrome, including stem elongation (Franklin and Quail, 2010).

Previous studies demonstrated a reduction in total leaf area in tomato and cucumber seedlings when exposed to increased blue light (Yousef et al., 2021; Hamedalla et al., 2022). In this study, under the B and B+UV-A treatments, the total leaf area decreased in tomato seedlings but did not change in cucumber seedlings (**Figure 2G**). These might be because that the cucumber varieties used in this study (cv. Zaoqing No. 2) was relatively insensitive to blue light, resulting in different plant responses. Our results showed that the total leaf area of tomato seedlings in the UV-A treatment was not significantly different from that in the control (**Figure 2G**). These results were not in line with the result on tomato that supplementation of UV-A (daily UV-A dose > 1.17 kJ-m^–2^ · d^–1^) increased the total leaf area (Kang et al., 2018), but the dose of UV-A light used in the study was different from our study. Under the R3B7+ UV-A treatment, the total leaf area of cucumber seedlings was evidently increased, while that in the R5B5+ UV-A treatment showed no comparable difference (Jeong et al., 2020). In this study, the total leaf area of cucumber seedlings in the 3W2R+ UV-A treatment did not differ from that of those in the control (3W2R treatment) (**Figure 2G**). These different effects of light quality on leaf development in these treatments might be due to the different light quality ratios or combinations. Interestingly, in our study, in cucumber seedlings under the far-red light supplement treatments (FR, UV-A+FR, and B+FR), the total leaf area was elevated only in the FR treatment (**Figure 2G**), suggesting that there might be an antagonism effect between far-red light and blue light or UV-A light on leaf development. To adapt to the low R:FR ratio caused by FR radiation, plants increased their total leaf area to harvest more light, but this hinged on species and growth conditions (Demotes-Mainard et al., 2016). Total leaf area has been considered a key parameter in determining plant growth (Weraduwage et al., 2015). Our Pearson analysis showed that the seedling index was positively correlated with the total leaf area in cucumber seedlings (**Figure 5B**). Among all treatments in cucumber, only the FR treatment showed a significant increase in the seedling index, and the total leaf area merely increased in the FR treatment (**Figure 5G**; **Table 1**). The highest total leaf area observed in the FR treatment might be attributed to the low R: FR radiation inducing rapid leaf development, resulting in increased leaf area and a large accumulation of photosynthetic products in the leaves (Park and Runkle, 2017).

The fresh and dry weights of the “Oxheart” tomato seedlings were increased, whereas those of the “Cherry” and “Roma” tomato seedlings were unaffected by adding UV-A light (Mariz-Ponte et al., 2018). Supplementation with far-red light and 25B (blue light) increased the total dry weight of the tomato plants, whereas 50–100B had no effect (Liang et al., 2021; Wang et al., 2021). Here, our data suggested that there was no obvious discrepancy in the dry weight of tomato seedlings among all the treatments (**Figure 3A**). These results demonstrated that the influence of supplemental light of different qualities on plant biomass is jointly determined by light quality, light intensity, and variety. The accumulation of plant dry matter is also affected by the total leaf area, since the light energy intercepted by plants will increase with the increase in total leaf area (Park and Runkle, 2018). In this paper, the total leaf area of tomato seedlings treated with UV-A, FR, UV-A+FR, and B+FR had no significant difference compared with the control (**Figure 2G**), which corresponds to the result that there was no significant difference in the total dry weight and the shoot dry weight between these four treatments and the control (**Figure 3A**). In the results of Pearson analysis and the PCA of cucumber seedlings, the seedling index was positively correlated with the plant dry weight, the shoot dry weight, and the root dry weight (**Figure 5B**). Under the FR treatment, the increase in total leaf area of cucumber seedlings may promote a marked increase in plant dry weight and shoot dry weight, thus enhancing the seedling index (**Figures 2G**, **3B**).

With respect to a single growth index, like plant height, stem diameter, and biomass, comprehensive indexes, such as compactness and the seedling index, can more comprehensively reflect the overall growth quality of seedlings (Bai et al., 2014; Vu et al., 2014). Usually, the higher the seedling index and compactness, the higher the quality of the seedlings (Bai et al., 2014; An et al., 2020). Previous studies have revealed that more compact pepper and tomato seedlings were produced by appropriately increasing blue light (Hernández et al., 2016; Liu et al., 2022). In addition, exposure to UV-A increased the compactness of cucumber and pepper seedlings (Jeong et al., 2020; Liu et al., 2022). Consistently, the compactness of cucumber and tomato seedlings under B and B+UV-A treatments significantly increased (**Table 1**). In the UV-A treatment, being distinct from the increased compactness of cucumber seedlings, those of tomato seedlings did not differ from the control (**Table 1**). Since plant compactness represents the ratio of shoot dry weight to plant height, the unaffected shoot dry weight and plant height of tomato seedlings in the UV-A treatment might explain why there is no significant difference in compactness between the UV-A treatment and control (**Table 1**). The compactness of both cucumber and tomato seedlings grown under FR, B+FR, and UV-A+FR treatments significantly decreased, which might be due to the promotion of far-red light on the seedling height. According to the results of Pearson analysis and PCA in tomato seedlings, the seedling index was significantly positively correlated with compactness (**Figure 5A**). The increased seedling index and compactness of the B+UV-A treatment meant that the light quality of this treatment was advantageous for cultivating high-quality tomato seedlings. What’s interesting is that although the seedling height, stem diameter, total leaf area, dry weight, fresh weight, the seedling index, compactness, and photosynthetic pigment content of tomato seedlings under the UV-A treatment were not significantly greater than those of the control, the comprehensive score of tomato seedlings obtained by comprehensive analysis in the UV-A treatment was higher than that in control, which indicated that the light condition of the UV-A treatment was more suitable for cultivating tomato seedlings. These results might be due to the relatively small component contribution value of each growth index in the comprehensive formula (**Supplementary Table S2** **)**. In cucumber, the seedling height, stem diameter, total leaf area, whole dry weight, shoot dry weight, and the seedling index of cucumber seedlings under FR treatment significantly increased. Correspondingly, the comprehensive analysis in this study showed that FR treatment was the most favourable lighting condition for cultivating high-quality cucumber seedlings.

Furthermore, the results of the Pearson analysis indicated that the seedling growth difference between the B, UV-A, B+UV-B, and CK treatments and the FR, UV-A+FR, and B+FR treatments, no matter in tomato or cucumber, was small **(** **Figures 6A, B**). These suggested that the effects of different light qualities on different plants were similar to some extent; undoubtedly, more experiments should be conducted in the future to clarify these differences.

### Effects of different light qualities on photosynthetic characteristics and photosynthetic pigments of tomato and cucumber seedlings

UV-A did not cause an evident influence on the chlorophyll content of cucumber and tomato plants (Brazaitytė et al., 2009; Brazaitytė et al., 2010), similar results in the UV-A and B+UV-A treatments were also observed in this work (**Table 2**). Supplementation of far-red light decreased the chlorophyll content in both tomato and cucumber plants (Wang et al., 2021). Our FR treatment did not affect the chlorophyll content of tomatoes and cucumbers, while the FR+B treatments reduced the chlorophyll content of tomatoes and the carotenoid content of cucumbers, respectively. Adding blue light promoted chlorophyll accumulation in cucumber (Hernández and Kubota, 2016). In this study, a stimulation of chlorophyll contents was only detected in the B treatment when supplemented with blue light. In tomato, the contents of chlorophyll a, chlorophyll b, and total chlorophyll were not affected in the B, UV-A, and FR treatments, whereas these significantly reduced in the UV-A+FR and B+FR treatments (**Table 2**). These results collectively suggested that the effect of different light spectral combinations on plant photosynthetic pigments was a complex response process, which might be a consequence of the interaction between different light spectra.

Fv/Fm represents the potential quantum efficiency of PSII; the smaller the value, the greater the photoinhibition of plants (Kumar et al., 2014). Generally, in normally growing plants, the maximum quantum yield of PSII (Fv/Fm value) is approximately 0.83 (Björkman and Demmig, 1987). The Fv/Fm values of seedlings in this study are approximately 0.78 (**Table 2**), which might be a result of the incomplete light spectral range of the used LED lights. UV radiation induces photosynthetic protein degradation, causing negative stress on the PSII (Greenberg et al., 1989). However, compared to the control, no significant differences in the Fv/Fm values were exhibited in tomato and cucumber seedlings under UV-A light (UV-A, B+UV-A, and UV-A+FR treatments) (**Table 2**), indicating that these seedlings were not under light stress. In tomato seedlings, the Fv/Fm values in the FR treatment were not significantly different from those in the control (**Table 2**); similar results were found in a previous report (Wang et al., 2021). In cucumber seedlings, the Fv/Fm were unaffected in the B treatment but remarkably decreased in the FR treatment in comparison with the control (**Table 2**), implying that the FR treatment led to inhibition of PSII in cucumber leaves. Surprisingly, the Fv/Fm values of cucumber seedlings in the B+FR treatment were significantly greater than those in the control (**Table 2**), which means that blue light in the B+FR treatment could alleviate the inhibition of PSII caused by far-red light, but the interaction between blue and far-red light needs further investigation.

In the six light treatments except CK, both the Y (II) and ETR values significantly reduced in tomato seedlings but markedly enhanced in cucumber seedlings (**Table 2**). These indicated that the actual quantum yield and electron transfer rate of the control were comparatively higher in tomato but lower in cucumber (Yousef et al., 2021). In cucumber seedlings, although the chlorophyll content was unaffected in the B+FR treatment, the photosynthesis rate significantly increased in the B+FR treatment (**Table 2**), which might be caused by the difference in leaf microstructures (Yao et al., 2017).

### Effects of different light qualities on antioxidant system of tomato and cucumber seedlings

The antioxidant enzyme activity in plant tissues, which are affected by different light spectrums, clearly states a valid response to various stresses. Cucumber plants that radiated under blue light exhibited higher activities of SOD and CAT, with increased tolerance to Cd stress (Guo et al., 2022). Tomato seedlings supplemented with far-red light had elevated activities of SOD, POD, and CAT and enhanced salt resistance (Wang et al., 2021). However, in this study, in comparison with the control, the activity of SOD and POD decreased in the FR and UV-A+FR treatments of tomato seedlings (**Figures 4B, C**), which might be related to differences in the background light quality or the plant cultivars, causing different experimental results.

After being exposed to blue light, the content of chlorophyll and soluble protein in cucumber increased, which will indirectly balance the active oxygen in the species, thus increasing the activity of antioxidant enzymes (Wang et al., 2009). In this study, the increase in the content of chlorophyll and soluble protein in the B treatment might be contributed to the increased activities of SOD and POD in cucumber seedlings (**Figures 4B, C**). In contrast to the control seedlings, cucumber seedlings under the UV-A and B+UV-A treatments had significantly elevated activities of SOD and POD, with significantly decreased MDA content in the B+UV-A treatment (**Figures 4B–E**), suggesting that supplementation of blue and UV-A light alone and UV-A+B was beneficial to increasing the antioxidant level of cucumber seedlings, which needs further demonstration.

### Effects of different light qualities on the growth and development of tomato and cucumber after transplanting

Our results showed that the flowering time was delayed significantly in cucumber seedlings grown under the FR treatments, but it was not affected in tomato plants (**Table 3**). Supplementation of FR radiation at the seedling stage had no effect on the flowering time of geranium (Park and Runkle, 2017). From these, the effect of far-red light radiation on the flowering of different species and its mechanism deserves further exploration. From the PCA results, in tomato, there was no significant correlation between the flowering time and measured physiological indices before transplanting (**Figure 5A**). In cucumber, the flowering time was positively correlated with Fv/Fm **(** **Figures 5B**, **6B** **)**, which was in agreement with the results that Fv/Fm was significantly reduced and the flowering time was significantly delayed under the FR treatment. Certain correlations exist between the vegetative and reproductive growth of plants (Cremer, 1992). In the FR treatment, the decrease in Fv/Fm might result in insufficient nutrient growth, which may lead to the delay of flowering time in cucumber plants.

For cucumber, the node position of the first female flower in seedlings treated with FR treatment was lower (**Table 4**), indicating that adding far-red light alone could promote the formation of female flowers. It is known that the sexual differentiation of cucumber is affected by hormones such as ethylene and gibberellin (Zhang et al., 2017). In addition, far-red light can affect the synthesis of plant hormones (Islam et al., 2014). In this study, far-red light might influence female flower differentiation by affecting the synthesis of hormones. Despite that the total flower number was obviously unaffected within 20 DAT in the B, UV-A, and FR treatments, this was significantly reduced in the UV-A+FR and B+FR treatments (**Table 4**), implying that supplementing with UV-A+FR or B+FR at the seedling stage appears to be not good for flower development in cucumber plants. This phenomenon and its mechanism deserve in-depth research in the future.

## Conclusion

This study investigated the growth of tomato and cucumber seedlings under different light treatments (CK, B, UV-A, FR, B+UV-A, UV-A+FR, and FR+BB+FR) and the development of these seedlings after transplanting. There are some similarities in the growth morphology of the two varieties under different lighting environments: the growth morphology was promoted under the FR, UV-A+FR, and B+FR treatments but was inhibited under the B treatment. The B+UV-A treatment and the FR treatment increased the seedling index of tomato and cucumber seedlings, respectively. The B treatment increased the chlorophyll content, Y (II), and ETR of cucumber. In the UV-A treatment, the activities of SOD and POD were repressed in tomatoes but enhanced in cucumbers. The UV-A and FR treatments were beneficial for the flower development of tomato and cucumber after transplantation, respectively. In the future, the correlation between light quality and environmental factors such as photoperiod and temperature can be conducted through multiple variables, in order to obtain a better environment for seedling growth and provide a reference for the development of the vegetable seedling industry in plant factories and greenhouses.

## Data availability statement

The original contributions presented in the study are included in the article/**Supplementary Material.** Further inquiries can be directed to the corresponding author.

## Author contributions

XL, RS, and MG performed the experiments and wrote the manuscript. RH and YL performed the experiments and statistical analyses. HL conceived and designed the experiments. HL acquired of funding and contributed to revised the manuscript. All authors have read and agreed to the published version of the manuscript.
