# Supplementary material for: Growth of tomato and cucumber seedlings under different light environments and their development after transplanting
Source: Front Plant Sci. 2023 Jul 18;14:1164768. doi: 10.3389/fpls.2023.1164768 (PMC10400448; doi:10.3389/fpls.2023.1164768)
Supplement: Supplementary file 1 [file Table_1.docx]

Supplementary Material

Growth of tomato and cucumber seedlings under different light environments and their development after transplanting

Xiaojuan Liu ^1^, Rui Shi ^1^, Meifang Gao ^1^, Rui He ^1^, Yamin Li ^1^, Houcheng Liu ^1^*

*** Correspondence:** Houcheng Liu: liuhch@scau.edu.cn

# Supplementary Figures and Tables

## Supplementary Tables

| PPFD (μmolm^-2^ s^-1^) | CK | B | UV-A | FR | B+  UV-A | UV-A+  FR | B+FR |
| --- | --- | --- | --- | --- | --- | --- | --- |
| PPFD (400~700 nm) | 251.33 | 262.71 | 255.55 | 255.88 | 259.69 | 253.25 | 256.17 |
| UV-A(380~399nm) | 0.07 | 0.24 | 7.09 | 0.12 | 6.59 | 6.56 | 0.26 |
| Blue (400~499 nm) | 45.21 | 91.05 | 46.21 | 44.79 | 93.15 | 45.57 | 88.288 |
| Green (500~599 nm) | 69.95 | 58.47 | 70.34 | 70.59 | 57.60 | 69.8 | 58.62 |
| Red (600~699 nm) | 136.16 | 113.19 | 139 | 140.49 | 108.93 | 137.87 | 109.26 |
| Far red (700~800 nm) | 3.99 | 3.53 | 3.80 | 28.117 | 5.52 | 32.17 | 29.22 |
| YPFD（400~700 nm) | 222.38 | 224.27 | 229.71 | 229.34 | 221.81 | 230.67 | 219.4 |
| R:FR | 34.1 | 32.04 | 36.58 | 5.00 | 19.73 | 4.28 | 3.73 |
| R:B | 3.01 | 1.24 | 3.01 | 3.14 | 1.17 | 3.03 | 1.24 |

**Table S1.** Spectral data of various light treatments of tomato and cucumber seedlings.

The two principal components PC1 and PC2 with the largest difference contribution rate of principal components were extracted. The eigen values of the first two principal components were all greater than 1, and the cumulative variance contribution rate was 57.63% (Table 2.1). All index data were standardized (Z_n_ represents standardized data, N represents index).

The variance contribution rate of each principal component was taken, and the weighted average of the principal components was carried out to construct a comprehensive score model for evaluating the growth and development of pepper seedlings with different light quality as

Y=32.78% Y1+24.86% Y2.

**Table S2.1** Principal component analysis results of tomato seedlings.

| Component scoring coefficient matrix | | | | | |
| --- | --- | --- | --- | --- | --- |
| Indicator | Y1 | Y2 | Indicator | Y1 | Y2 |
| Seedling height | -0.27 | -0.07 | Total chlorophyll | 0.16 | 0.18 |
| Stem diameter | -0.28 | 0.01 | Carotenoid content | -0.16 | -0.18 |
| Hypocotyl | -0.22 | -0.06 | Fv/Fm | 0.06 | 0.04 |
| True leaf number | -0.08 | 0.21 | Y(II) | 0.13 | -0.01 |
| Whole fresh weight | -0.26 | 0.16 | ETR | 0.11 | -0.02 |
| Root fresh weight | -0.20 | 0.16 | SOD activity | 0.23 | 0.10 |
| Shoot fresh weight | -0.26 | 0.15 | POD activity | 0.13 | 0.11 |
| Whole dry weight | -0.16 | 0.29 | CAT activity | 0.18 | 0.13 |
| Root dry weight | -0.09 | 0.26 | Soluble protein content | 0.24 | 0.11 |
| Shoot dry weight | -0.17 | 0.28 | MDA content | 0.25 | 0.08 |
| Compactness | 0.17 | 0.25 | Time of the first flower | -0.05 | -0.03 |
| Root shoot ratio | 0.14 | -0.05 | The node position of the first flower | -0.09 | -0.07 |
| Specific leaf weight | 0.14 | 0.09 | Number of fruits on the 15th day | 0.13 | -0.08 |
| Percentage of shoot dry matter | 0.22 | 0.16 | Number of fruits on the 30th day | 0.13 | -0.02 |
| The seedling index | 0.06 | 0.30 |  |  |  |
| chlorophyll a | 0.16 | 0.18 |  |  |  |
| chlorophyll b | 0.16 | 0.18 |  |  |  |
| Variance  contribution % | | 32.78 (Y1) | | 24.86 (Y2) | |

| Treatment | Y1 | Y2 | total | rank |
| --- | --- | --- | --- | --- |
| CK | 0.40 | -0.05 | 0.35 | 3 |
| B | 0.55 | -0.12 | 0.43 | 2 |
| UV-A | 0.02 | 0.42 | 0.45 | 1 |
| FR | -0.16 | -0.16 | -0.32 | 5 |
| B+UV-A | 0.16 | 0.06 | 0.22 | 4 |
| UV-A+FR | -0.48 | -0.11 | -0.59 | 7 |
| B+FR | -0.26 | -0.12 | -0.38 | 6 |

**Table S2.2** The score of each principal component and total score of each treatment.

The two principal components PC1 and PC2 with the largest difference contribution rate of principal components were extracted. The eigen values of the first two principal components were all greater than 1, and the cumulative variance contribution rate was 50.46% (Table 3.1). All index data were standardized (Z_n_ represents standardized data, N represents index).

The variance contribution rate of each principal component was taken, and the weighted average of the principal components was carried out to construct a comprehensive score model for evaluating the growth and development of pepper seedlings with different light quality as

Y=35.23% Y1+15.23% Y2.

**Table S3.1** Principal component analysis results of tomato seedlings.

| Component scoring coefficient matrix | | | | | |
| --- | --- | --- | --- | --- | --- |
| Indicator | Y1 | Y2 | Indicator | Y1 | Y2 |
| Seedling height | 0.23 | -0.21 | Total chlorophyll | -0.01 | 0.04 |
| Stem diameter | 0.27 | -0.04 | Carotenoid content | 0.03 | 0.03 |
| Hypocotyl | 0.11 | -0.32 | Fv/Fm | -0.01 | -0.13 |
| True leaf number | 0.15 | -0.09 | Y(II) | 0.06 | -0.07 |
| Whole fresh weight | 0.27 | 0.02 | ETR | 0.06 | -0.07 |
| Root fresh weight | 0.22 | 0.15 | SOD activity | -0.15 | 0.32 |
| Shoot fresh weight | 0.27 | -0.05 | POD activity | 0.04 | -0.01 |
| Whole dry weight | 0.26 | 0.14 | CAT activity | -0.04 | 0.15 |
| Root dry weight | 0.23 | 0.16 | Soluble protein content | -0.19 | 0.26 |
| Shoot dry weight | 0.26 | 0.14 | MDA content | 0.16 | 0.04 |
| Compactness | -0.07 | 0.38 | Time of the first flower | -0.03 | -0.11 |
| Root shoot ratio | -0.18 | -0.09 | The node of the first flower | 0.00 | -0.04 |
| Specific leaf weight | 0.06 | 0.03 | Number of flowers within the 15 nodes | -0.06 | 0.14 |
| Percentage of shoot dry matter | -0.01 | 0.38 | Number of female flowers within the 15 nodes | -0.03 | -0.06 |
| The seedling index | 0.19 | 0.28 | The node of the first female flower | -0.05 | 0.09 |
| chlorophyll a | 0.00 | 0.05 |  |  |  |
| chlorophyll b | -0.02 | 0.01 |  |  |  |
| Variance  contribution % | | 35.23 (Y1) | | 15.23 (Y2) | |

**Table S3.2** Each principal component score and total score of each treatment

| Treatment | Y1 | Y2 | total | rank |
| --- | --- | --- | --- | --- |
| CK | -0.21 | -0.20 | -0.01 | 7 |
| B | -0.07 | -0.20 | 0.13 | 3 |
| UV-A | -0.16 | -0.25 | 0.09 | 5 |
| FR | 0.75 | 0.64 | 0.11 | 1 |
| B+UV-A | -0.10 | -0.19 | 0.09 | 4 |
| UV-A+FR | -0.03 | 0.20 | -0.23 | 2 |
| B+FR | -0.19 | 0.00 | -0.19 | 6 |
